# Understanding the Landscape of Cancer Care in Europe: Evaluating Clinical and Comprehensive Cancer Centers

**DOI:** 10.3390/healthcare12232338

**Published:** 2024-11-22

**Authors:** Denis Horgan, Marc Van den Bulcke, Núria Malats, Ruggero de Maria, France Dube, Jaya Singh, Paul Hofman, Muhammad Imran Omar, Umberto Malapelle, Tanya Hills, Francesco Pepe, Vivek Subbiah

**Affiliations:** 1 European Alliance for Personalised Medicine, 1040 Brussels, Belgium; jayasinghtec29@gmail.com; 2 Department of Molecular and Cellular Engineering, Jacob Institute of Biotechnology and Bioengineering, Faculty of Engineering and Technology, Sam Higginbottom University of Agriculture, Technology and Sciences, Prayagraj 211007, India; 3 Belgian Cancer Centre, Sciensano, 1050 Brussels, Belgium; marc.vandenbulcke@sciensano.be; 4 Genetic and Molecular Epidemiology Group, Spanish National Cancer Research Centre (CNIO), 28029 Madrid, Spain; 5 Institute of General Pathology, Catholic University of the Sacred Heart, 20123 Rome, Italy; presidenza@alleanzacontroilcancro.it; 6 Astra Zeneca, Concord Pike, Wilmington, DE 19803, USA; france.dube@astrazeneca.com; 7 Laboratory of Clinical and Experimental Pathology, IHU RespirERA, FHU-OncoAge, Côte d’Azur University, 06000 Nice, France; hofman.p@chu-nice.fr; 8 European Association of Urology, Research Fellow Academic Urology Unit, University of Aberdeen, Aberdeen AB25 2ZD, UK; m.i.omar@abdn.ac.uk; 9 Department of Public Health, University Federico II of Naples, 80138 Naples, Italy; umberto.malapelle@unina.it (U.M.); francesco.pepe4@unina.it (F.P.); 10 Boehringer Ingelheim International GmbH, 55218 Ingelheim am Rhein, Germany; tanya.hills@boehringer-ingelheim.com; 11 Sarah Cannon Research Institute, Nashville, TN 37203, USA

**Keywords:** clinical cancer centers, comprehensive cancer centers, healthcare delivery, oncological care, innovation in healthcare

## Abstract

Background: A comparison of the operations of Clinical Cancer Centers and Comprehensive Cancer Centers across Europe provides novel data on the interrelation between different factors in care delivery. Method: The analysis is based on a survey of key dimensions in care delivery, comparing routine treatment, advanced technology integration, research participation, and innovation adoption across the two types of centers. Results: Clinical Cancer Centers excel in providing routine cancer treatment through multidisciplinary teams but struggle with advanced technology integration and research participation. In contrast, Comprehensive Cancer Centers offer robust infrastructure and focus on research, advanced diagnostics, and innovative therapies, yet they face challenges in fully integrating these technologies into patient care. Conclusion: Collaboration between the two types of centers could enhance overall cancer care effectiveness, leveraging the routine efficiency of Clinical Centers and the innovative capabilities of Comprehensive Centers. By addressing gaps in technology adoption, supportive care integration, and research involvement, a more holistic cancer care network can be established, ensuring that patients across Europe access both foundational care and the latest therapeutic options.

## 1. Introduction

Cancer stands as one of the most formidable challenges confronting global public health, exerting a profound impact on individuals, families, and healthcare systems worldwide. In Europe, where healthcare systems strive for excellence and equity, the management of cancer represents a complex interplay of scientific advancements, clinical practice, and healthcare policy [1]. At the forefront of this battle are Clinical and Comprehensive Cancer Centers that integrate cutting-edge research with multidisciplinary care [2].

The landscape of cancer care in Europe is characterized by a blend of remarkable progress and persistent challenges. Advances in understanding cancer biology, coupled with the development of targeted therapies and precision medicine approaches, have markedly improved treatment outcomes and survival rates over recent decades [3]. However, these advancements have not uniformly benefited all patients across Europe. Disparities persist in access to specialized care, treatment outcomes, and survival rates among different demographic groups and geographical regions. Factors such as socioeconomic status, healthcare infrastructure, and regional variations in healthcare policy contribute to these inequities [4].

In 2023, the European Union introduced a new directive as part of its ongoing strategy to control cancer, further strengthening the continent’s commitment to combating this disease. The 2023 directive emphasizes the need for enhanced coordination among member states, improved data sharing, and the development of new standards for cancer care delivery across Europe. It also underscores the importance of reducing inequalities in cancer care and ensuring that all EU citizens have access to the same high-quality treatments and preventive measures, regardless of their location.

Clinical and Comprehensive Cancer Centers play a pivotal role in mitigating these disparities by offering specialized expertise, state-of-the-art technologies, and multidisciplinary care essential for delivering high-quality cancer treatment and management. These centers serve as hubs of innovation, where clinical trials, translational research, and personalized medicine approaches converge to advance the frontiers of cancer care. Beyond treatment, these centers also significantly contribute to cancer prevention, early detection, survivorship care, and palliative support [5].

The operation framework of Clinical and Comprehensive Cancer Centers is designed to align with and support the EU’s strategy to combat cancer, functioning as central nodes in a network that ensure the consistent application of best practices across Europe. Moreover, these centers serve as catalysts for the integration of research into clinical practice, fostering a collaborative environment where healthcare professionals, researchers, and industry partners synergistically translate scientific discoveries into tangible patient benefits. They play a crucial role in educating and training healthcare professionals, ensuring the continuous development of expertise in oncology and related disciplines. As centers of excellence, they attract talent and investment, driving economic growth and innovation within their respective regions. The continuous education and training of healthcare professionals ensure that the latest advances in cancer care are rapidly adopted into clinical practice [6].

However, the extent to which these centers advance the EU’s new policy on controlling cancer varies. While many Clinical and Comprehensive Cancer Centers are well-positioned to implement the 2023 directive by serving as models of best practice, contributing to the standardization of care and leading in innovative research, challenges remain. Some centers may face difficulties in aligning fully with the new directive due to resource constraints, regional disparities, or varying levels of integration within the broader EU framework. Ensuring that all centers can contribute to and benefit from the 2023 directive will require ongoing efforts to harmonize practices, enhance infrastructure, and foster collaboration across borders.

The evaluation of Clinical and Comprehensive Cancer Centers is crucial for optimizing their contribution to healthcare systems and improving patient outcomes. Assessments encompass various dimensions, including adherence to clinical guidelines, patient safety standards, service delivery efficiency, research productivity, and patient-reported outcomes. By systematically evaluating these aspects, healthcare stakeholders can identify areas for improvement, allocate resources effectively, and implement evidence-based practices that enhance the quality and efficiency of cancer care delivery. Systematic evaluations help to uncover best practices and areas where improvements are needed, guiding policy decisions and resource allocation [7].

This study aims to undertake a comprehensive evaluation of Clinical and Comprehensive Cancer Centers across diverse geographical regions in Europe. By evaluating their capabilities and impact on patient outcomes, the study aims to inform evidence-based strategies for enhancing cancer care delivery, improving survival rates, and ultimately, advancing the quality of life for cancer patients across Europe and beyond.

## 2. Methodology

Key pillars and measures were defined through a comprehensive process involving expert consultations and a review of the existing literature to assess critical aspects of Cancer Centers, encompassing Clinical Services, Research and Education, Technology and Innovation, Laboratory Infrastructure, Clinical Trials, Patient Care, and Performance Metrics. These foundational elements guided the development of structured surveys tailored to each type of cancer center, such as those specializing in lung cancer, breast cancer, and prostate cancer.

The surveys were designed based on the pillars and measures to capture quantitative data relevant to each pillar (Table 1).

The survey was distributed across Europe, targeting cancer centers that are part of the European Society for Medical Oncology (ESMO) or affiliated with other recognized medical organizations. The centers were identified based on their association with these organizations, ensuring they represent a broad spectrum of expertise and geographical locations. The survey was conducted between April 2024 and June 2024 and only targeted the Comprehensive Cancer Centers and Clinical Cancer Centers. The survey questions can be found in the Supplementary Materials. 

Data were collected through a structured questionnaire distributed to a sample of cancer centers across Europe. Out of 60 centers initially approached, 43 responded, with 36 responses deemed valid for analysis. This included contributions from 23 Comprehensive Cancer Centers and 13 Clinical Cancer Centers. Each center was rated on a 1–5 point scale scoring system, where higher scores indicated better performance or higher capability in each respective pillar and measure; then, the data were transformed into z-scores to facilitate comparative analysis. The z-scores were calculated for the entire sample, ensuring equal weight was assigned to each cancer center across both categories: Comprehensive Cancer Centers and Clinical Cancer Centers. Z-scores provided a standardized measure of each center’s performance relative to the mean, allowing identification of areas where centers were performing above or below the average.

The z-score analysis served as a sensitivity check by standardizing the data, enabling objective comparisons across centers. It highlighted specific strengths and areas needing improvement while ensuring consistency in the interpretation of results. By focusing solely on z-score analysis, the study ensured a straightforward and standardized approach to evaluate performance, focusing on each pillar’s contribution to overall cancer care quality and research output. This methodology provided a clear, quantitative framework for assessing and benchmarking cancer center capabilities across the Europe.

### 2.1. Comprehensive Cancer Centre

The Z-Score analysis across various pillars of the Comprehensive Cancer Centers reveals the following key insights:

#### 2.1.1. Clinical Services

The Clinical Services pillar indicates both strengths and areas for improvement. Comprehensive Cancer Centers scored positively for the availability of multidisciplinary teams (0.36), highlighting a strong foundation for collaborative care. This setup is crucial in managing complex cancer cases where input from various specialists can lead to improved treatment outcomes. Access to palliative care services also had a positive score (0.11), demonstrating a moderate focus on end-of-life care, which is essential for patient comfort and quality of life. However, there are notable challenges in the integration of supportive care services (−0.24) and the availability of specialized cancer clinics (−0.31). These areas reflect potential gaps in the comprehensive and specialized support systems, which could limit the accessibility and effectiveness of care for patients requiring targeted treatment.

#### 2.1.2. Research and Education

The Research and Education pillar highlights considerable gaps in infrastructure and participation. Most measures showed negative scores, with a particularly low score for participation in cancer research networks (−0.61). This suggests that Comprehensive Cancer Centers may lack adequate support and resources to engage actively in cancer research initiatives. Research infrastructure availability (−0.34) and availability of fellowship and residency programs (−0.20) also scored low, indicating limited support for training the next generation of oncology professionals and researchers. These findings suggest a potential talent and resource gap in research activities, which could hinder advancements in clinical practices and innovation within these centers.

#### 2.1.3. Technology and Innovation

This pillar revealed some of the most significant challenges, particularly in adopting advanced technologies crucial for personalized cancer treatment. The use of telemedicine for cancer care (−0.68) and the implementation of precision medicine (−0.66) had some of the lowest scores, suggesting that centers may be lagging in technology integration. The limited use of AI in diagnostics (−0.22) further points to slow adoption of technologies that could improve diagnostic accuracy and efficiency. The findings indicate a considerable need for investment in technology and innovation to align Comprehensive Cancer Centers with the evolving landscape of precision oncology.

#### 2.1.4. Laboratory Infrastructure

The Laboratory Infrastructure pillar presents a more positive picture, especially in terms of basic laboratory equipment availability (0.50), indicating that Comprehensive Cancer Centers are generally well-equipped for routine laboratory procedures. Access to specialized laboratory services, including high-throughput sequencing (0.04) and pathology services (0.09), also scored positively, though modestly. This suggests that while basic laboratory infrastructure is robust, there remains room for improvement in advanced molecular diagnostics, which are essential for personalized treatment planning.

#### 2.1.5. Research and Development

Comprehensive Cancer Centers appear well-positioned in Research and Development, with high scores in the capacity for basic cancer research (0.49) and collaboration with other research institutions (0.37). These strengths indicate that the centers are fostering a productive research environment and building partnerships to further cancer research. However, the low score for the availability of research grants and funding (0.07) highlights potential financial constraints that could limit the scope and sustainability of research projects, affecting long-term innovation.

#### 2.1.6. Clinical Trials

The Clinical Trials pillar demonstrates moderate readiness, with a positive score for trial infrastructure (0.22), underscoring a foundational capability to conduct clinical trials, a critical component of advancing cancer treatments. However, negative scores for access to experimental therapies (−0.07) and availability of clinical trial coordinators (−0.10) suggest limitations in resources needed to manage and expand clinical trial programs effectively. These gaps may impede patients’ access to emerging therapies and reduce the potential for innovative treatment options.

#### 2.1.7. Patient Care

Patient Care scored well overall, particularly for the availability of psychosocial support services (0.33) and patient navigation (0.19). These services are essential for holistic cancer care, addressing patients’ emotional needs and guiding them through complex treatment processes. The presence of specialized cancer care units scored positively (0.07), although modestly, indicating that centers have some specialization but could further enhance targeted support for specific cancer types.

#### 2.1.8. Performance Metrics

The Performance Metrics pillar emphasizes a patient-centered approach, with patient satisfaction scores (0.33) and treatment outcome metrics (0.06) scoring positively. This shows a commitment to evaluating patient-reported outcomes and treatment efficacy. However, there is variability in the utilization of predictive metrics, such as sensitivity (−0.08), specificity (0.07), PPV (−0.08), and NPV (−0.09), which could affect the precision of diagnostics. Strengthening the use of these metrics could improve diagnostic accuracy and patient care.

#### 2.1.9. Subset Analysis Based on Therapy Type

The scores in this pillar reveal challenges in screening and trial methodologies, especially for immuno-oncology (IO) therapies, with pre-screening challenges scoring −0.52. This indicates that Comprehensive Cancer Centers face significant difficulties in implementing effective screening processes for these advanced therapies. However, a slight positive score for pan-cancer trial methodologies (0.04) suggests some capacity for multi-cancer research, although additional support may be needed to optimize this approach.

#### 2.1.10. Comparative Analysis and Evaluation

In Comparative Analysis and Evaluation, there is a strong emphasis on practical application, with high scores for the practical implications of screening methodologies (0.33). This shows that Comprehensive Cancer Centers are attentive to the real-world effectiveness of diagnostic methods, which can directly impact patient outcomes. However, the slightly negative score (−0.07) for assessing advantages and limitations of each screening methodology suggests that centers may need to improve their evaluative processes to ensure optimal diagnostic strategies.

#### 2.1.11. Diagnostic Test Interpretation

The Diagnostic Test Interpretation pillar indicates a preference for team-based interpretation, with positive scores for interpretation by a dedicated team (0.18) and through a consultation process (0.17). This collaborative approach is likely to enhance diagnostic precision. On the other hand, a negative score (−0.22) for individual specialist interpretation suggests that these centers may limit reliance on single-specialist evaluations, possibly due to the complex nature of cancer diagnostics.

#### 2.1.12. Communication to Healthcare Providers and Patients

Communication practices at Comprehensive Cancer Centers are varied, with a preference for direct consultation and patient portals. Positive scores were noted for the combination of provider consultation and patient portal (0.20), indicating a focus on ensuring patients receive information through multiple, accessible channels. Mixed scores for communication with healthcare providers, such as electronic communication (0.18) and printed reports (0.05), suggest a hybrid approach. This could enhance coordination, but standardization may be needed for consistent information sharing.

#### 2.1.13. Turnaround Time for Results

Turnaround Time for Results showed moderate efficiency, with positive scores for IHC report turnaround time (0.20) and small panel NGS report time (0.05). These results indicate a relatively timely approach to providing essential diagnostic information. However, the slightly negative score for liquid biopsy report times (−0.08) could signal delays in this area, which may impact treatment planning for patients requiring rapid results.

#### 2.1.14. Incorporation into Treatment Planning

The results for Incorporation into Treatment Planning underscore a strong reliance on multidisciplinary team discussions, which scored positively (0.36), supporting a comprehensive approach to decision-making in cancer treatment. Additional positive scores for oncologist-guided treatment (0.18) and patient involvement (0.08) demonstrate an inclusive approach to treatment planning, ensuring that patient perspectives are valued alongside clinical insights.

These scores collectively highlight both strengths and gaps in Comprehensive Cancer Centers’ capabilities, identifying areas where targeted improvements could significantly enhance cancer care delivery across Europe (Table 2).

### 2.2. Clinical Cancer Centre

The Z-Score analysis across various pillars of the Clinical Cancer Centers reveals the following key insights:

#### 2.2.1. Clinical Services

In the Clinical Services pillar, Clinical Cancer Centers show a notable strength in the availability of multidisciplinary teams (0.26), underscoring a well-rounded approach to patient care that incorporates multiple specialist perspectives. However, there is room for improvement in the integration of supportive care services (−0.25), which could indicate a need for more cohesive and accessible support resources for patients undergoing treatment. The availability of specialized cancer clinics (0.07) and access to palliative care services (0.13) are moderate, reflecting that while these services are present, there is potential to enhance access and support for end-of-life care and specialized interventions.

#### 2.2.2. Research and Education

The Research and Education pillar reveals a mixed picture. Clinical Cancer Centers demonstrate strength in the availability of education and training programs (0.29), which could contribute to developing a skilled workforce. However, challenges remain with research infrastructure availability (−0.18), participation in cancer research networks (−0.15), and availability of fellowship and residency programs (−0.05), all of which indicate limitations in research capacity and professional development opportunities. These gaps suggest that while educational foundations are in place, further support is necessary to foster robust research environments.

#### 2.2.3. Technology and Innovation

Clinical Cancer Centers face substantial challenges in the Technology and Innovation pillar, with negative scores across several measures. The lowest score, for the implementation of precision medicine (−0.56), points to a significant gap in personalized treatment approaches. Similar limitations are observed in the use of telemedicine for cancer care (−0.39) and the implementation of AI in diagnostics (−0.35), suggesting slow adoption of modern technological advancements. Availability of advanced treatment technologies (−0.17) also scored low, indicating a need for investment in innovative tools and systems to enhance treatment precision and accessibility.

#### 2.2.4. Laboratory Infrastructure

Laboratory Infrastructure shows some positive aspects, particularly in the availability of basic laboratory equipment (0.31), suggesting that Clinical Cancer Centers are adequately equipped for routine procedures. However, scores for access to specialized laboratory services (−0.05), availability of high-throughput sequencing (−0.09), and availability of pathology services (−0.04) indicate areas where advanced diagnostics and specialized testing capabilities are limited. Strengthening these resources could support more comprehensive diagnostic services, particularly for personalized treatment planning.

#### 2.2.5. Research and Development

Clinical Cancer Centers exhibit strengths in Research and Development, with positive scores in capacity for basic cancer research (0.42) and collaboration with other research institutions (0.20). These results highlight active engagement in foundational research and cross-institutional partnerships. However, a lower score for participation in translational research (−0.08) and a modest score for research grants and funding availability (0.06) indicate that financial and collaborative support for applied research could be bolstered, particularly in bridging the gap between laboratory findings and clinical application.

#### 2.2.6. Clinical Trials

Clinical Cancer Centers have a moderately positive foundation in Clinical Trials, with infrastructure for conducting trials (0.17) and access to experimental therapies (0.07) indicating readiness to offer patients access to emerging treatments. However, the availability of clinical trial coordinators (−0.16) and participation in trials (0.05) reflect gaps in personnel resources, which could limit the scale and effectiveness of trial programs. Addressing these shortages could enhance the centers’ capacity to conduct a broader range of clinical trials.

#### 2.2.7. Patient Care

In Patient Care, Clinical Cancer Centers show moderate scores in the availability of specialized cancer care units (0.15), patient navigation and support services (0.04), patient education programs (0.13), and psychosocial support services (0.07). These results suggest a balanced but somewhat basic approach to patient support, with each service available but perhaps not extensively developed. Strengthening these services, particularly psychosocial support, could further enhance the holistic care approach for cancer patients.

#### 2.2.8. Performance Metrics

The Performance Metrics pillar shows variation, with some metrics like patient satisfaction scores (0.16) indicating that patients are generally content with their care. However, the use of sensitivity (−0.15), specificity (0.05), PPV (−0.07), and NPV (−0.20) as metrics presents challenges in diagnostic accuracy and reliability. Improving these predictive metrics could enhance the precision of diagnostic assessments, ultimately benefiting patient outcomes.

#### 2.2.9. Subset Analysis Based on Therapy Type

Challenges are particularly notable in the subset analysis for therapy types, especially in pre-screening challenges for IO therapies (−0.31), suggesting a need for more robust methodologies in pre-screening patients for immuno-oncology therapies. Scores for screening approaches for high-prevalence biomarkers (0.06) and strategies for rare cancer types (−0.16) reflect a basic capacity, though further refinement is needed. The positive score for methodologies in pan-cancer trials (0.14) shows potential for broader multi-cancer research, albeit with a need for increased specificity.

#### 2.2.10. Comparative Analysis and Evaluation

Clinical Cancer Centers perform moderately well in Comparative Analysis and Evaluation, with strengths in practical implications of screening methodologies (0.30) and a balanced view of each methodology’s advantages and limitations (0.16). These scores suggest that centers are attentive to practical application in diagnostics and treatments, ensuring that chosen methodologies have clear, beneficial outcomes for patients.

#### 2.2.11. Diagnostic Test Interpretation

The Diagnostic Test Interpretation pillar reflects a collaborative approach, with positive scores for interpretation by a dedicated team (0.03) and through consultation (0.07). This structure can improve diagnostic accuracy by incorporating diverse perspectives. However, interpretation by individual specialists (−0.08) and other methods (−0.17) show variability in practice, suggesting that enhancing standardized processes could further support diagnostic consistency.

#### 2.2.12. Communication to Healthcare Providers and Patients

The Communication pillar reflects diverse methods, with positive scores for electronic communication (0.05), direct consultation (0.08), and combined approaches for provider–patient communication (0.13). Printed reports (−0.06) and some alternative methods (−0.41) scored lower, suggesting that while direct and digital communications are prioritized, traditional methods may be less effective or timely. A balanced and standardized approach to communication could improve consistency and information flow.

#### 2.2.13. Turnaround Time for Results

Turnaround Time for Results shows moderate efficiency, with positive scores in IHC report turnaround (0.07) and NGS report times for small panels (0.13), indicating responsiveness in delivering diagnostic information. However, the negative score for large-panel NGS (−0.16) and slightly low liquid biopsy turnaround (0.01) suggest areas for improvement, particularly in complex diagnostics that may require longer processing times.

#### 2.2.14. Incorporation into Treatment Planning

Incorporation into Treatment Planning reveals strengths in multidisciplinary discussion of results (0.26) and oncologist-led guidance for treatment (0.14). Patient involvement in treatment discussions (0.08) reflects an inclusive approach, although lower scores for alternative methods (−0.08) may indicate reliance on traditional frameworks. Enhancing flexibility in incorporating varied patient preferences could further personalize treatment planning.

This comprehensive overview reveals both strong areas and specific opportunities for improvement across Clinical Cancer Centers, particularly in technology adoption, research support, and diagnostic methodologies, which can inform targeted strategies for elevating cancer care across European regions. (Table 3).

## 3. Discussion

In comparing Comprehensive Cancer Centers and Clinical Cancer Centers across various pillars, we observe distinct strengths and challenges that reflect their unique roles in the healthcare system. Each center type brings specific assets and faces particular limitations, suggesting that a complementary approach could maximize patient outcomes and research impact.

### 3.1. Clinical Cancer Center

Clinical Cancer Centers play a critical role in providing frontline cancer care, with strengths in essential services such as the availability of multidisciplinary teams and specialized cancer clinics. These centers are integral in ensuring that patients have access to core cancer treatments, supportive care, and palliative services, as reflected in the moderately positive scores for clinical services. However, a significant gap is apparent in the integration of supportive care services, which could enhance patient quality of life if further developed. Additionally, the negative scores in the Technology and Innovation pillar reveal that Clinical Cancer Centers struggle to adopt and implement advanced technologies like precision medicine, telemedicine, and AI in diagnostics. This suggests a reliance on more conventional treatment approaches, potentially limiting access to cutting-edge therapies [8].

Clinical Cancer Centers also face challenges in research infrastructure, as indicated by negative scores in Research and Education and Research and Development. Although they provide some foundational education and training programs, participation in cancer research networks and the availability of research grants and funding are limited. These constraints could restrict the centers’ ability to attract and retain specialized staff, reducing their overall research output. On the diagnostic side, Clinical Cancer Centers have demonstrated a need for improved laboratory infrastructure, especially in terms of high-throughput sequencing and access to specialized services. Enhancing these capabilities could enable these centers to better support personalized treatment planning through accurate biomarker identification and rapid diagnostic turnaround times [9].

Overall, Clinical Cancer Centers demonstrate a robust capacity for routine cancer care and education but lack the advanced technology and research capabilities that are hallmarks of Comprehensive Cancer Centers. Addressing these gaps, particularly in supportive care integration, technological innovation, and research involvement, could help Clinical Cancer Centers play a more dynamic role in the evolving cancer care landscape.

### 3.2. Comprehensive Cancer Center

Comprehensive Cancer Centers, on the other hand, display more advanced capabilities across several pillars, especially in research, technology, and patient care. Their stronger infrastructure in Research and Education highlights a focus on academic and clinical research, contributing to knowledge generation and high-level training. The positive scores in the availability of education and training programs, research infrastructure, and collaboration with other research institutions underscore their active role in pushing forward cancer science. These centers also have a greater capacity for translational research, bridging laboratory findings with clinical applications. This advantage in research and development highlights the Comprehensive Cancer Centers’ ability to implement innovative treatments and contribute to scientific advancements in cancer care [5].

Technology and Innovation scores for Comprehensive Cancer Centers reflect a stronger adoption of advanced technologies, though gaps remain. Precision medicine, telemedicine, and AI in diagnostics are being implemented, albeit with challenges. As Comprehensive Cancer Centers often have the funding and institutional support to experiment with new technologies, they are generally better positioned to offer patients access to cutting-edge therapies and experimental treatments. However, negative scores in some technology measures indicate that even Comprehensive Cancer Centers face obstacles in fully integrating these technologies into patient care. Addressing these gaps could ensure that patients receive the full benefits of precision and personalized medicine, particularly for rare and complex cancers [10].

Comprehensive Cancer Centers also excel in Clinical Trials, showing a strong infrastructure for conducting trials and access to experimental therapies. This is crucial for advancing treatment options and offering patients access to new therapies that are not yet widely available. Moreover, these centers perform well in patient care, offering specialized cancer units, navigation services, and psychosocial support. This comprehensive approach to patient care highlights their commitment to addressing the multifaceted needs of cancer patients, including mental and emotional support alongside physical treatment [11].

In terms of diagnostic capabilities, Comprehensive Cancer Centers benefit from more extensive laboratory resources, which enable them to conduct high-throughput sequencing, pathology, and other advanced diagnostic techniques. This ensures that patients receive timely and accurate diagnoses, which are critical for personalized treatment planning. The positive scores in laboratory infrastructure and diagnostic test interpretation reflect these centers’ capacity to handle complex cases with precision [12].

### 3.3. Comparative Analysis and Synthesis

In synthesizing these findings, it becomes clear that Comprehensive Cancer Centers offer a more resource-intensive, research-focused approach to cancer care, benefiting from robust infrastructure, technological capabilities, and a multidisciplinary treatment environment. Clinical Cancer Centers, while effective in providing routine care and essential services, often lack the resources for advanced research and technology implementation. This suggests an opportunity for stronger collaborations between Comprehensive and Clinical Cancer Centers to maximize impact across the spectrum of cancer care. By aligning their complementary strengths—routine care efficiency in Clinical Centers and advanced research and innovation in Comprehensive Centers—patients could benefit from improved accessibility to both foundational care and cutting-edge treatments [13].

Furthermore, targeted investments in Clinical Cancer Centers could address some of their critical gaps, particularly in supportive care integration, technology adoption, and research involvement. Expanding access to advanced diagnostics and training Clinical Cancer Centers in implementing new technologies could help bridge the gap between routine care and specialized research-driven care. Conversely, enhancing patient-centric services in Comprehensive Cancer Centers could foster a more holistic approach to care, ensuring that high-level research does not come at the expense of personalized, supportive care [14].

Ultimately, both Clinical and Comprehensive Cancer Centers play crucial roles in the cancer care ecosystem. A balanced approach that leverages the operational strengths of Clinical Centers and the innovative potential of Comprehensive Centers could create a more integrated and effective cancer care network, fostering improved patient outcomes and advancing the field of oncology in Europe.

## 4. Conclusions

This assessment highlights the complementary roles of Comprehensive Cancer Centers and Clinical Cancer Centers in the cancer care landscape. While Clinical Cancer Centers excel in delivering routine cancer care and essential patient services, Comprehensive Cancer Centers are better equipped for cutting-edge research, advanced diagnostics, and the integration of innovative technologies. Each type of center has its unique strengths and areas for improvement, which, if addressed, could foster a more balanced and effective approach to cancer care. Clinical Cancer Centers benefit patients by providing accessible, high-quality care in a structured and supportive environment, whereas Comprehensive Cancer Centers contribute significantly to advancements in oncology through research and specialized treatments. By strategically aligning these strengths, the overall cancer care system could be enhanced, ensuring that patients across Europe have access to both foundational care and the latest therapeutic options [15].

## Figures and Tables

**Table 1:** List of pillars, measures, and variable number.

SR. No.	Pillars	Measures	Variable No.
1	Clinical Services	Availability of Multidisciplinary Teams	V1
	Clinical Services	Integration of Supportive Care Services	V2
	Clinical Services	Availability of Specialized Cancer Clinics	V3
	Clinical Services	Access to Palliative Care Services	V4
2	Research and Education	Research Infrastructure Availability	V5
	Research and Education	Availability of Education and Training Programs	V6
	Research and Education	Participation in Cancer Research Networks	V7
	Research and Education	Availability of Fellowship and Residency Programs	V8
3	Technology and Innovation	Availability of Advanced Treatment Technologies	V9
	Technology and Innovation	Implementation of Precision Medicine	V10
	Technology and Innovation	Use of Telemedicine for Cancer Care	V11
	Technology and Innovation	Implementation of AI in Diagnostics	V12
4	Laboratory Infrastructure	Basic Laboratory Equipment Availability	V13
	Laboratory Infrastructure	Access to Specialized Laboratory Services	V14
	Laboratory Infrastructure	Availability of High-throughput Sequencing	V15
	Laboratory Infrastructure	Availability of Pathology Services	V16
5	Research and Development	Capacity for Basic Cancer Research	V17
	Research and Development	Collaboration with Other Research Institutions	V18
	Research and Development	Availability of Research Grants and Funding	V19
	Research and Development	Participation in Translational Research	V20
6	Clinical Trials	Participation in Clinical Trials	V21
	Clinical Trials	Access to Experimental Therapies	V22
	Clinical Trials	Infrastructure for Conducting Clinical Trials	V23
	Clinical Trials	Availability of Clinical Trial Coordinators	V24
7	Patient Care	Availability of Specialized Cancer Care Units	V25
	Patient Care	Patient Navigation and Support Services	V26
	Patient Care	Patient Education Programs	V27
	Patient Care	Availability of Psychosocial Support Services	V28
8	Performance Metrics	Use of Sensitivity as a Metric	V29
	Performance Metrics	Use of Specificity as a Metric	V30
	Performance Metrics	Use of Positive Predictive Value (PPV) as a Metric	V31
	Performance Metrics	Use of Negative Predictive Value (NPV) as a Metric	V32
	Performance Metrics	Timing of Patient Eligibility Assessment	V33
	Performance Metrics	Patient Satisfaction Scores	V34
	Performance Metrics	Treatment Outcome Metrics	V35
9	Subset Analysis Based on Therapy Type	Pre-screening Challenges for IO Therapies	V36
	Subset Analysis Based on Therapy Type	Screening Approaches for High Prevalence Biomarkers	V37
	Subset Analysis Based on Therapy Type	Strategies for Rare Cancer Types	V38
	Subset Analysis Based on Therapy Type	Methodologies for Pan Cancer Trials	V39
10	Comparative Analysis and Evaluation	Advantages and Limitations of Each Screening Methodology	V40
	Comparative Analysis and Evaluation	Practical Implications of Screening Methodologies	V41
11	Diagnostic Test Interpretation	Interpretation by a Dedicated Team	V42
	Diagnostic Test Interpretation	Interpretation by Individual Specialists	V43
	Diagnostic Test Interpretation	Interpretation Through Consultation Process	V44
	Diagnostic Test Interpretation	Other Methods of Interpretation (please specify)	V45
12	Communication to Healthcare Providers	Electronic Communication Through HIS	V46
	Communication to Healthcare Providers	Printed Reports Delivered to Physician’s Office	V47
	Communication to Healthcare Providers	Both Electronic and Printed Reports	V48
	Communication to Healthcare Providers	Other Methods of Communication (please specify)	V49
	Communication to Patients	Direct Communication During Consultation	V50
	Communication to Patients	Through a Dedicated Patient Portal	V51
	Communication to Patients	Combination of Provider Consultation and Patient Portal	V52
	Communication to Patients	Other Methods of Communication (please specify)	V53
13	Turnaround Time for Results	Pathology Report Turnaround Time	V54
	Turnaround Time for Results	IHC Report Turnaround Time	V55
	Turnaround Time for Results	NGS Report Turnaround Time (Small Panel)	V56
	Turnaround Time for Results	NGS Report Turnaround Time (Large Panel)	V57
	Turnaround Time for Results	Liquid Biopsy Report Turnaround Time	V58
14	Incorporation into Treatment Planning	Results Discussed by Multidisciplinary Team	V59
	Incorporation into Treatment Planning	Oncologist Uses Results to Guide Treatment	V60
	Incorporation into Treatment Planning	Patient Involvement in Treatment Discussions	V61
	Incorporation into Treatment Planning	Other Methods of Incorporation (please specify)	V62

**Table 2:** Descriptive statistics and overall Z-score rating for Comprehensive Cancer Centers.

**Variable No.**	**Mean**	**Standard Error**	**Median**	**Mode**	**Standard Deviation**	**Sample Variance**	**Range**	**Minimum**	**Maximum**	**Count**
V1	3.80	0.39	4.00	5.00	1.23	1.51	3	2	5	23
V2	3.40	0.31	3.00	3.00	0.97	0.93	3	2	5	23
V3	3.30	0.45	3.00	5.00	1.42	2.01	4	1	5	23
V4	3.60	0.43	3.50	5.00	1.35	1.82	3	2	5	23
V5	3.30	0.26	3.50	4.00	0.82	0.68	2	2	4	23
V6	3.50	0.34	3.50	4.00	1.08	1.17	3	2	5	23
V7	3.10	0.31	3.00	4.00	0.99	0.99	3	1	4	23
V8	3.40	0.37	3.50	4.00	1.17	1.38	3	2	5	23
V9	3.30	0.33	3.50	4.00	1.06	1.12	3	2	5	23
V10	3.10	0.31	3.00	3.00	0.99	0.99	3	2	5	23
V11	3.10	0.18	3.00	3.00	0.57	0.32	2	2	4	23
V12	3.40	0.22	3.50	4.00	0.70	0.49	2	2	4	23
V13	3.90	0.28	4.00	3.00	0.88	0.77	2	3	5	23
V14	3.60	0.31	3.50	3.00	0.97	0.93	3	2	5	23
V15	3.60	0.27	4.00	4.00	0.84	0.71	3	2	5	23
V16	3.60	0.31	3.50	3.00	0.97	0.93	3	2	5	23
V17	3.90	0.23	4.00	4.00	0.74	0.54	2	3	5	23
V18	3.80	0.25	4.00	3.00	0.79	0.62	2	3	5	23
V19	3.60	0.16	4.00	4.00	0.52	0.27	1	3	4	23
V20	3.50	0.34	3.50	4.00	1.08	1.17	3	2	5	23
V21	3.50	0.27	3.50	3.00	0.85	0.72	3	2	5	23
V22	3.50	0.31	3.00	3.00	0.97	0.94	3	2	5	23
V23	3.70	0.26	4.00	4.00	0.82	0.68	3	2	5	23
V24	3.50	0.27	3.50	4.00	0.85	0.72	3	2	5	23
V25	3.60	0.31	3.50	3.00	0.97	0.93	3	2	5	23
V26	3.70	0.21	4.00	4.00	0.67	0.46	2	3	5	23
V27	3.60	0.31	3.50	3.00	0.97	0.93	3	2	5	23
V28	3.80	0.25	4.00	4.00	0.79	0.62	2	3	5	23
V29	3.50	0.22	4.00	4.00	0.71	0.50	2	2	4	23
V30	3.60	0.22	3.50	3.00	0.70	0.49	2	3	5	23
V31	3.50	0.22	4.00	4.00	0.71	0.50	2	2	4	23
V32	3.50	0.22	3.00	3.00	0.71	0.50	2	3	5	23
V33	3.60	0.16	4.00	4.00	0.52	0.27	1	3	4	23
V34	3.80	0.25	4.00	4.00	0.79	0.62	2	3	5	23
V35	3.60	0.16	4.00	4.00	0.52	0.27	1	3	4	23
V36	3.20	0.25	3.00	3.00	0.79	0.62	2	2	4	23
V37	3.60	0.34	4.00	4.00	1.07	1.16	3	2	5	23
V38	3.40	0.27	3.00	3.00	0.84	0.71	3	2	5	23
V39	3.60	0.27	4.00	4.00	0.84	0.71	3	2	5	23
V40	3.50	0.27	3.50	4.00	0.85	0.72	3	2	5	23
V41	3.80	0.20	4.00	4.00	0.63	0.40	2	3	5	23
V42	3.70	0.21	4.00	4.00	0.67	0.46	2	3	5	23
V43	3.40	0.22	3.50	4.00	0.70	0.49	2	2	4	23
V44	3.70	0.21	4.00	4.00	0.67	0.46	2	3	5	23
V45	3.50	0.22	4.00	4.00	0.71	0.50	2	2	4	23
V46	3.50	0.27	3.50	4.00	0.85	0.72	3	2	5	23
V47	3.60	0.22	3.50	3.00	0.70	0.49	2	3	5	23
V48	3.70	0.21	4.00	4.00	0.67	0.46	2	3	5	23
V49	3.30	0.21	3.00	3.00	0.67	0.46	2	2	4	23
V50	3.70	0.21	4.00	4.00	0.67	0.46	2	3	5	23
V51	3.60	0.22	3.50	3.00	0.70	0.49	2	3	5	23
V52	3.70	0.21	4.00	4.00	0.67	0.46	2	3	5	23
V53	3.50	0.17	3.50	4.00	0.53	0.28	1	3	4	23
V54	3.60	0.22	3.50	3.00	0.70	0.49	2	3	5	23
V55	3.70	0.26	3.50	3.00	0.82	0.68	2	3	5	23
V56	3.60	0.16	4.00	4.00	0.52	0.27	1	3	4	23
V57	3.50	0.17	3.50	3.00	0.53	0.28	1	3	4	23
V58	3.50	0.22	3.00	3.00	0.71	0.50	2	3	5	23
V59	3.80	0.29	3.50	3.00	0.92	0.84	2	3	5	23
V60	3.70	0.21	4.00	4.00	0.67	0.46	2	3	5	23
V61	3.60	0.16	4.00	4.00	0.52	0.27	1	3	4	23
V62	3.60	0.16	4.00	4.00	0.52	0.27	1	3	4	23
**Variable No.**					**Overall Z-Score for Comprehensive** **Cancer** **Centre**					
V1					0.36					
V2					−0.24					
V3					−0.31					
V4					0.11					
V5					−0.34					
V6					−0.04					
V7					−0.61					
V8					−0.20					
V9					−0.35					
V10					−0.66					
V11					−0.68					
V12					−0.22					
V13					0.50					
V14					0.08					
V15					0.04					
V16					0.09					
V17					0.49					
V18					0.37					
V19					0.07					
V20					−0.08					
V21					−0.06					
V22					−0.07					
V23					0.22					
V24					−0.10					
V25					0.07					
V26					0.19					
V27					0.09					
V28					0.33					
V29					−0.08					
V30					0.07					
V31					−0.08					
V32					−0.09					
V33					0.05					
V34					0.33					
V35					0.06					
V36					−0.52					
V37					0.11					
V38					−0.22					
V39					0.04					
V40					−0.07					
V41					0.33					
V42					0.18					
V43					−0.22					
V44					0.17					
V45					−0.08					
V46					−0.07					
V47					0.05					
V48					0.18					
V49					−0.38					
V50					0.22					
V51					0.05					
V52					0.20					
V53					−0.08					
V54					0.07					
V55					0.20					
V56					0.05					
V57					−0.09					
V58					−0.08					
V59					0.36					
V60					0.18					
V61					0.08					
V62					0.03					

**Table 3:** Descriptive statistics and overall Z-Score rating for Clinical Cancer Centers.

**Variable No.**	**Mean**	**Standard Error**	**Median**	**Mode**	**Standard Deviation**	**Sample Variance**	**Range**	**Minimum**	**Maximum**	**Count**
V1	3.69	0.33	4.00	4.00	1.18	1.40	3	2	5	13
V2	3.31	0.26	3.00	3.00	0.95	0.90	3	2	5	13
V3	3.54	0.37	4.00	5.00	1.33	1.77	4	1	5	13
V4	3.54	0.37	3.00	5.00	1.33	1.77	3	2	5	13
V5	3.38	0.27	4.00	4.00	0.96	0.92	3	2	5	13
V6	3.69	0.29	4.00	4.00	1.03	1.06	3	2	5	13
V7	3.46	0.31	4.00	4.00	1.13	1.27	4	1	5	13
V8	3.46	0.31	3.00	3.00	1.13	1.27	3	2	5	13
V9	3.38	0.29	3.00	3.00	1.04	1.09	3	2	5	13
V10	3.15	0.25	3.00	3.00	0.90	0.81	3	2	5	13
V11	3.31	0.21	3.00	3.00	0.75	0.56	3	2	5	13
V12	3.23	0.26	3.00	3.00	0.93	0.86	3	2	5	13
V13	3.69	0.26	4.00	3.00	0.95	0.90	3	2	5	13
V14	3.46	0.27	3.00	3.00	0.97	0.94	3	2	5	13
V15	3.46	0.24	4.00	4.00	0.88	0.77	3	2	5	13
V16	3.46	0.27	3.00	3.00	0.97	0.94	3	2	5	13
V17	3.77	0.26	4.00	4.00	0.93	0.86	3	2	5	13
V18	3.62	0.24	4.00	3.00	0.87	0.76	3	2	5	13
V19	3.54	0.22	4.00	4.00	0.78	0.60	3	2	5	13
V20	3.46	0.29	4.00	4.00	1.05	1.10	3	2	5	13
V21	3.54	0.27	4.00	4.00	0.97	0.94	3	2	5	13
V22	3.54	0.27	3.00	3.00	0.97	0.94	3	2	5	13
V23	3.62	0.27	4.00	4.00	0.96	0.92	3	2	5	13
V24	3.38	0.27	3.00	3.00	0.96	0.92	3	2	5	13
V25	3.62	0.24	4.00	3.00	0.87	0.76	3	2	5	13
V26	3.54	0.22	4.00	4.00	0.78	0.60	3	2	5	13
V27	3.62	0.24	4.00	4.00	0.87	0.76	3	2	5	13
V28	3.54	0.24	3.00	3.00	0.88	0.77	3	2	5	13
V29	3.38	0.24	3.00	3.00	0.87	0.76	3	2	5	13
V30	3.54	0.22	4.00	4.00	0.78	0.60	3	2	5	13
V31	3.46	0.24	4.00	4.00	0.88	0.77	3	2	5	13
V32	3.38	0.21	3.00	3.00	0.77	0.59	3	2	5	13
V33	3.54	0.22	4.00	4.00	0.78	0.60	3	2	5	13
V34	3.62	0.24	4.00	3.00	0.87	0.76	3	2	5	13
V35	3.46	0.22	3.00	3.00	0.78	0.60	3	2	5	13
V36	3.31	0.24	3.00	3.00	0.85	0.73	3	2	5	13
V37	3.54	0.29	4.00	4.00	1.05	1.10	3	2	5	13
V38	3.38	0.27	3.00	3.00	0.96	0.92	3	2	5	13
V39	3.62	0.27	4.00	4.00	0.96	0.92	3	2	5	13
V40	3.62	0.24	4.00	3.00	0.87	0.76	3	2	5	13
V41	3.69	0.24	4.00	4.00	0.85	0.73	3	2	5	13
V42	3.54	0.22	4.00	4.00	0.78	0.60	3	2	5	13
V43	3.46	0.24	4.00	4.00	0.88	0.77	3	2	5	13
V44	3.54	0.24	3.00	3.00	0.88	0.77	3	2	5	13
V45	3.38	0.24	3.00	3.00	0.87	0.76	3	2	5	13
V46	3.54	0.27	4.00	4.00	0.97	0.94	3	2	5	13
V47	3.46	0.24	3.00	3.00	0.88	0.77	3	2	5	13
V48	3.54	0.22	4.00	4.00	0.78	0.60	3	2	5	13
V49	3.23	0.23	3.00	3.00	0.83	0.69	3	2	5	13
V50	3.54	0.22	4.00	4.00	0.78	0.60	3	2	5	13
V51	3.54	0.24	3.00	3.00	0.88	0.77	3	2	5	13
V52	3.62	0.24	4.00	4.00	0.87	0.76	3	2	5	13
V53	3.46	0.22	3.00	3.00	0.78	0.60	3	2	5	13
V54	3.54	0.22	4.00	4.00	0.78	0.60	3	2	5	13
V55	3.54	0.24	3.00	3.00	0.88	0.77	3	2	5	13
V56	3.62	0.21	4.00	4.00	0.77	0.59	3	2	5	13
V57	3.38	0.21	3.00	3.00	0.77	0.59	3	2	5	13
V58	3.54	0.24	3.00	3.00	0.88	0.77	3	2	5	13
V59	3.69	0.26	4.00	3.00	0.95	0.90	3	2	5	13
V60	3.62	0.21	4.00	4.00	0.77	0.59	3	2	5	13
V61	3.54	0.22	4.00	4.00	0.78	0.60	3	2	5	13
V62	3.46	0.22	3.00	3.00	0.78	0.60	3	2	5	13
**Variable No.**			**Overall Z-Score for Clinical Cancer Centre**							
V1			0.26							
V2			−0.25							
V3			0.07							
V4			0.13							
V5			−0.18							
V6			0.29							
V7			−0.15							
V8			−0.05							
V9			−0.17							
V10			−0.56							
V11			−0.39							
V12			−0.35							
V13			0.31							
V14			−0.05							
V15			−0.09							
V16			−0.04							
V17			0.42							
V18			0.20							
V19			0.06							
V20			−0.08							
V21			0.05							
V22			0.07							
V23			0.17							
V24			−0.16							
V25			0.15							
V26			0.04							
V27			0.13							
V28			0.07							
V29			−0.15							
V30			0.05							
V31			−0.07							
V32			−0.20							
V33			0.04							
V34			0.16							
V35			−0.05							
V36			−0.31							
V37			0.06							
V38			−0.16							
V39			0.14							
V40			0.16							
V41			0.30							
V42			0.03							
V43			−0.08							
V44			0.07							
V45			−0.17							
V46			0.05							
V47			−0.06							
V48			0.02							
V49			−0.41							
V50			0.08							
V51			0.03							
V52			0.13							
V53			−0.02							
V54			0.03							
V55			0.07							
V56			0.13							
V57			−0.16							
V58			0.01							
V59			0.26							
V60			0.14							
V61			0.08							
V62			−0.08							

## Data Availability

All data generated or analyzed during this study are included in this published article.

## Supplementary Materials

The following supporting information can be downloaded at https://www.mdpi.com/article/10.3390/healthcare12232338/s1: Survey S1: Cancer Center Capacity Survey.
