# Supplementary material for: Understanding the Landscape of Cancer Care in Europe: Evaluating Clinical and Comprehensive Cancer Centers
Source: Healthcare (Basel). 2024 Nov 22;12(23):2338. doi: 10.3390/healthcare12232338 (PMC11640883; doi:10.3390/healthcare12232338)
Supplement: Supplementary file 1 [file healthcare-12-02338-s001.zip › healthcare-3324661-supplementary.pdf]

Cancer Centre Capacity and Readiness Survey

Part 1: General Information

- Cancer center name: (Fill in)
- Location (city, state): (Fill in)
- Number of beds: (Fill in)
- Cancer center type:
  a. ( ) Comprehensive
  b. ( ) Basic
  c. ( ) Clinical
- Primary focus (e.g., adult oncology, pediatric oncology):
  a. ( ) Adult oncology
  b. ( ) Pediatric oncology
  c. ( ) Other (please specify): ___________

1. Clinical Services

Availability of multidisciplinary teams for cancer care:

(A) Not Available

(B) Limited Availability

(C) Available

(D) Well-Established

Integration of supportive care services (e.g., palliative care, psychosocial support):

(A) Not Available

(B) Limited Integration

(C) Partially Integrated

(D) Fully Integrated

Does your facility have specialized clinics for specific cancer types (e.g., breast, lung)?

a) Yes, for all major cancer types
b) Yes, for some cancer types
c) No, but plans to develop them
d) No specialized clinics

2. Research and Education

Research infrastructure (e.g., labs, equipment):

(A) Not Available

(B) Limited Availability

(C) Available

(D) Well-Established

Education and training programs for healthcare professionals:

(A) Not Available

(B) Limited Availability

(C) Available

(D) Well-Established

Is your facility part of a national or international cancer research network?

a) Yes, national network
b) Yes, international network
c) Yes, both national and international
d) No

How many oncology fellowship or residency positions are available at your center each year?

a) 1-5
b) 6-10
c) 11-20
d) More than 20
e) None

3. Technology and Innovation

Availability of advanced treatment technologies (e.g., radiotherapy, surgery):

(A) Not Available

(B) Limited Availability

(C) Available

(D) Well-Established

Implementation of innovative treatment approaches (e.g., precision medicine, immunotherapy):

(A) Not Available

(B) Limited Implementation

(C) Partially Implemented

(D) Fully Implemented

What stage of AI integration for diagnostics (e.g., pathology, radiology) is your facility at?

a) Fully implemented
b) Partially implemented
c) Being piloted
d) Not implemented

4. Laboratory Infrastructure

Availability of basic laboratory equipment:

(A) Not Available

(B) Limited Availability

(C) Available

(D) Well-Established

Access to specialized laboratory services (e.g., pathology, genetics):

(A) Not Available

(B) Limited Access

(C) Available

(D) Well-Established

Does your facility offer high-throughput sequencing for cancer diagnostics?

a) Yes, routinely
b) Yes, but only for specific cases
c) No, but available through external partnerships
d) No, not available

5. Research and Development

Capacity for basic cancer research (e.g., cell biology, molecular biology):

(A) Not Available

(B) Limited Capacity

(C) Available

(D) Well-Established

Collaboration with other research institutions:

(A) No Collaboration

(B) Limited Collaboration

(C) Some Collaboration

(D) Extensive Collaboration

How would you assess the availability of research grants and funding for cancer research at your institution?

a) Abundant (ample funding from multiple sources)
b) Sufficient (adequate funding to meet most research needs)
c) Limited (some funding available but not sufficient)
d) Inadequate (significant difficulty in securing funding)
e) No available funding

How active is your facility in participating in translational research (bridging laboratory findings with clinical applications)?

a) Highly active (frequent and robust involvement in translational projects)
b) Moderately active (some ongoing translational research)
c) Minimally active (limited involvement in translational efforts)
d) Not active (no current translational research activities)
e) Other (please specify)

6. Clinical Trials

Participation in clinical trials:

(A) Not Participating

(B) Limited Participation

(C) Active Participation

(D) Leading Participation

Access to experimental therapies:

(A) Not Available

(B) Limited Availability

(C) Available

(D) Well-Established

How would you rate your facility's infrastructure for conducting clinical trials (e.g., coordinators, data systems)?

a) Excellent
b) Good
c) Fair
d) Poor
e) No infrastructure for clinical trials

How many clinical trial coordinators are available in your facility?

a) 0-1
b) 2-3
c) 4-5
d) More than 5

7. Patient Care

Availability of specialized cancer care units (e.g., oncology ward, outpatient clinics):

(A) Not Available

(B) Limited Availability

(C) Available

(D) Well-Established

Patient navigation and support services:

(A) Not Available

(B) Limited Availability

(C) Available

(D) Well-Established

Are there dedicated patient education programs to help patients understand their treatment options?

a) Yes, for all patients
b) Yes, but only for specific patient groups
c) No, but being developed
d) No education programs

8. Performance Metrics and Assessment

Which performance metrics do you use to evaluate the effectiveness of pre-screening methodologies? (Select all that apply)

(A) Sensitivity

(B) Specificity

(C) Positive Predictive Value (PPV)

(D) Negative Predictive Value (NPV)

(E) Percentage Enrolled

When do you assess patient eligibility during the screening process?

(A) At the beginning of the patient workup

(B) During the patient workup

(C) At the time of study enrollment

(D) At multiple timepoints

9. Subset Analysis Based on Therapy Type

Which challenges do you encounter in pre-screening for Immuno-Oncology (IO) therapies? (Select all that apply)

(A) Identifying patients with specific biomarkers

(B) Managing immune-related adverse events

(C) Assessing response to immunotherapy

(D) Other (please specify)

What screening approaches do you use for trials targeting high prevalence biomarkers (>30%)?

(A) Genetic testing

(B) Biomarker assays

(C) Imaging techniques

(D) Other (please specify)

How does your facility approach screening and treatment for rare cancer types?

a) Participation in rare cancer networks
b) Collaboration with international research institutions
c) Special clinical trial initiatives
d) Limited availability of screening
e) Other (please specify)

What screening methodologies are used in pan-cancer trials conducted at your facility?

a) Tumor mutation burden (TMB)
b) Liquid biopsy
c) Multi-gene panels
d) Whole-exome sequencing (WES)
e) Other (please specify)

10. Comparative Analysis and Evaluation

How do you compare the performance and efficacy of different pre-screening methodologies? (Select all that apply)

(A) Manual vs. Automated vs. Hybrid Solutions

(B) Advantages and limitations of each methodology

(C) Practical implications of each methodology

(D) Other (please specify)

Part 2: Diagnostic Services

2.1. Laboratory Infrastructure

2.1.1 Basic Laboratory Equipment:

Which of the following automated laboratory equipment is available in your center? (Select all that apply)

( ) Automated blood cell counters

( ) Chemistry analyzers

( ) Coagulation analyzers

( ) Other (please specify): ___________

2.1.2 Specialized Laboratory Services:

Comprehensive Cancer Center:

Which of the following specialized laboratory services are offered by your center (in-house or outsourced)? (Select all that apply)

( ) Pathology

( ) Immunohistochemistry (IHC)

( ) Flow cytometry

( ) In situ hybridization (ISH)

( ) Molecular diagnostics (please specify which techniques): __________

( ) Other (please specify): __________

Basic Cancer Center:

Select the primary specialized laboratory service offered by your center (in-house or outsourced):

( ) Pathology

( ) Other (please specify): __________

Clinical Cancer Center:

Which of the following specialized laboratory services are essential for your clinical trials program (in-house or outsourced)? (Select all that apply)

( ) Pathology

( ) Immunohistochemistry (IHC)

( ) Flow cytometry

( ) Other (please specify): __________

2.2. Molecular Diagnostics

Comprehensive Cancer Center:

2.2.1 Next-Generation Sequencing (NGS):

Does your center have access to NGS platforms?

( ) Yes

( ) No

If yes, which of the following types of NGS platforms are available in your center? (Select all that apply)

( ) Whole-exome sequencing (WES)

( ) Targeted sequencing panels

( ) RNA sequencing

( ) Other (please specify): __________

2.2.2 Utilization of NGS:

How frequently is NGS utilized for cancer diagnosis and patient management in your center?

( ) Not used

( ) Rarely used (<10% of cases)

( ) Occasionally used (10-30% of cases)

( ) Frequently used (>30% of cases)

2.2.3 Liquid Biopsy Testing:

Does your center offer liquid biopsy testing?

( ) Yes

( ) No

If yes, which of the following types of liquid biopsy tests are available in your center? (Select all that apply)

( ) Cell-free circulating tumor DNA (ctDNA) analysis

( ) Circulating tumor cells (CTCs) analysis

( ) Other (please specify): __________

2.2.4 Utilization of Liquid Biopsy:

How frequently is liquid biopsy utilized for cancer diagnosis and monitoring in your center?

( ) Not used

( ) Rarely used (<10% of cases)

( ) Occasionally used (10-30% of cases)

( ) Frequently used (>30% of cases)

2.2.5 Other Molecular Diagnostic Techniques:

Which of the following other molecular diagnostic techniques are available in your center? (Select all that apply)

( ) Polymerase Chain Reaction (PCR)

( ) Fluorescence In Situ Hybridization (FISH)

( ) Quantitative PCR (qPCR)

( ) Pyrosequencing

( ) Methylation analysis

( ) Other (please specify): __________

Basic and Clinical Cancer Centers:

Please indicate your level of access to the following molecular diagnostic techniques (select one for each):

NGS:

( ) Not available

( ) Access through collaboration with another center

( ) Limited in-house availability

Liquid Biopsy:

( ) Not available

( ) Access through collaboration with another center

( ) Limited in-house availability (specify which types, e.g., ctDNA only)

Part 3: Integration of Diagnostics in Patient Care

3.1. Test Selection and Ordering: (Fill in a brief description of the process for selecting and ordering diagnostic tests).

3.2. Interpretation and Reporting of Results:

How are diagnostic test results interpreted in your center? (Select all that apply)

( ) By a dedicated team of molecular pathologists and oncologists.

( ) By individual specialists based on their area of expertise.

( ) Through a consultation process involving multiple healthcare professionals.

( ) Other (please specify): __________

How are diagnostic test results communicated to healthcare providers? (Select one)

( ) Electronically through the hospital information system (HIS).

( ) Printed reports delivered to the physician's office.

( ) Both electronic and printed reports are provided.

( ) Other (please specify): __________

How are diagnostic test results communicated to patients? (Select one)

( ) Directly by the healthcare provider during a consultation.

( ) Through a dedicated patient portal with test results and explanations.

( ) A combination of provider consultation and patient portal access.

( ) Other (please specify): __________

3.3. Utilization of Results for Treatment Decisions:

How are diagnostic test results incorporated into treatment planning meetings? (Select all that apply)

( ) Results are presented and discussed by the multidisciplinary team.

( ) Test results are used to guide treatment recommendations by the oncologist.

( ) Patients are involved in discussions about how test results influence treatment options.

( ) Other (please specify): __________

3.4. Challenges and Opportunities:

Briefly describe any challenges your center faces regarding the utilization of diagnostics in cancer care. (Fill in)

Briefly describe any opportunities you see for improving the integration of diagnostics into patient care. (Fill in)

Part 4: Additional Information

Please use this space to provide any additional information about your center's diagnostic capabilities and utilization that you feel is relevant. (Fill in)
